# Dural Metastasis of Prostatic Adenocarcinoma Mimicking Meningioma en Plaque: Case Report and Systematic Review

**DOI:** 10.7759/cureus.113801

**Published:** 2026-08-02

**Authors:** Ayanne Alves de Oliveira, Paulo Eduardo Albuquerque Zito Raffa, Jose Luis Quelho Filho, Leonardo Vieira Nunes, Alan Mayck Cavalcante de Oliveira Filho, Maria Gabriella Romão de Oliveira, Kairone Bezerra Costa, Vitor Afonso Favaretto, Eliane Milharcix Zanovelo, Carlos E D'Aglio Rocha

**Affiliations:** 1 Department of Neurosurgery, Faculdade de Medicina de São José do Rio Preto, São José do Rio Preto, BRA; 2 School of Medicine, Unifacisa University Center, Campina Grande, BRA; 3 Department of Pathology, Faculdade de Medicina de São José do Rio Preto, São José do Rio Preto, BRA

**Keywords:** case report, central nervous system metastasis, dural metastasis, meningioma en plaque, neurosurgery, prostate adenocarcinoma

## Abstract

Dural metastasis from prostate adenocarcinoma is an uncommon manifestation of advanced disease and may closely mimic primary dural tumors such as meningioma en plaque, making diagnosis particularly challenging. We report the case of a 63-year-old man with a history of treated prostate adenocarcinoma who presented with a three-month history of headache, anorexia, significant weight loss, and progressive mental confusion. Cranial computed tomography and magnetic resonance imaging demonstrated a large right frontotemporoparietal extra-axial lesion with imaging characteristics suggestive of meningioma en plaque. The patient underwent microsurgical resection because of progressive neurological deterioration, significant mass effect, and diagnostic uncertainty. Histopathological and immunohistochemical analyses confirmed dural metastasis of prostatic adenocarcinoma. The postoperative course was complicated by venous infarction, extensive intraparenchymal hemorrhage, and refractory intracranial hypertension. To better characterize this rare presentation, we performed a systematic review in accordance with the Preferred Reporting Items for Systematic Reviews and Meta-Analyses (PRISMA) guidelines. PubMed and MEDLINE databases were searched, and 19 studies comprising 44 patients met the inclusion criteria. This case highlights the importance of considering dural metastasis in the differential diagnosis of atypical dural-based lesions in patients with a history of prostate cancer. However, because the currently available evidence is limited predominantly to isolated case reports and small case series, conclusions regarding optimal management should be interpreted with caution, and larger multicenter studies are needed to better define the clinical characteristics and management of this rare entity.

## Introduction

Prostate cancer is one of the leading causes of cancer-related morbidity and mortality among men worldwide and remains one of the most frequently diagnosed malignancies in the male population [1]. Improvements in systemic therapies, including androgen deprivation therapy, chemotherapy, and novel hormonal agents, have significantly prolonged survival, leading to an increasing incidence of metastatic involvement at uncommon sites, including the central nervous system (CNS) [2,3]. Nevertheless, intracranial metastases remain rare, occurring in fewer than 0.6% of patients with prostate cancer [4].

Among intracranial manifestations, dural metastases are considerably less frequent than parenchymal metastases but represent an important diagnostic challenge because they often mimic primary dural neoplasms, particularly meningioma en plaque [2,5,6]. Dural involvement is thought to occur through hematogenous dissemination, retrograde spread via Batson's venous plexus, or direct extension from calvarial metastases. Once the dura is infiltrated, reactive fibrosis, diffuse dural thickening, adjacent bone involvement, and homogeneous contrast enhancement may produce radiological findings that closely resemble those observed in meningioma en plaque [5-8]. Consequently, distinguishing these entities based solely on neuroimaging is frequently difficult.

Patients commonly present with nonspecific neurological manifestations such as headache, cognitive impairment, seizures, focal neurological deficits, or symptoms related to intracranial hypertension [5-8]. Because these clinical and radiological findings substantially overlap with those of primary extra-axial tumors, definitive diagnosis generally relies on histopathological examination associated with immunohistochemical analysis. Recognition of this rare entity is clinically important because the differential diagnosis may influence surgical planning, pathological interpretation, subsequent oncological management, and prognostic counseling.

Despite the increasing number of published reports, the available evidence remains limited almost exclusively to isolated case reports and small case series. Consequently, the clinical spectrum, imaging characteristics, optimal management, and outcomes of dural metastases from prostate adenocarcinoma remain incompletely characterized.

Herein, we report a rare case of dural metastasis secondary to prostate adenocarcinoma diagnosed 10 years after treatment of the primary tumor, presenting with clinical and radiological findings highly suggestive of meningioma en plaque. To better contextualize this uncommon presentation, we also performed a systematic review of the published literature to summarize the currently available evidence and highlight the diagnostic challenges associated with this rare condition.

## Case presentation

A 63-year-old man with a history of prostate adenocarcinoma treated 10 years earlier at an outside institution was admitted with a three-month history of progressive headache, anorexia, approximately 17 kg of unintentional weight loss, and worsening mental confusion during the previous month. Information regarding the initial tumor stage, Gleason score, previous systemic therapies, and oncological follow-up was unavailable because treatment had been performed at another institution. On admission, the patient had a Glasgow Coma Scale (GCS) score of 14 (E4V4M6) and a Karnofsky Performance Status (KPS) score of 70. Neurological examination revealed mental confusion without focal neurological deficits. Muscle strength and sensory examination were preserved in all extremities, and extraocular movements were intact without cranial nerve palsies. The serum prostate-specific antigen (PSA) level was 1,020 ng/mL, consistent with advanced systemic disease. He had no previous history of intracranial disease or neurosurgical procedures.

**Figure 1:**
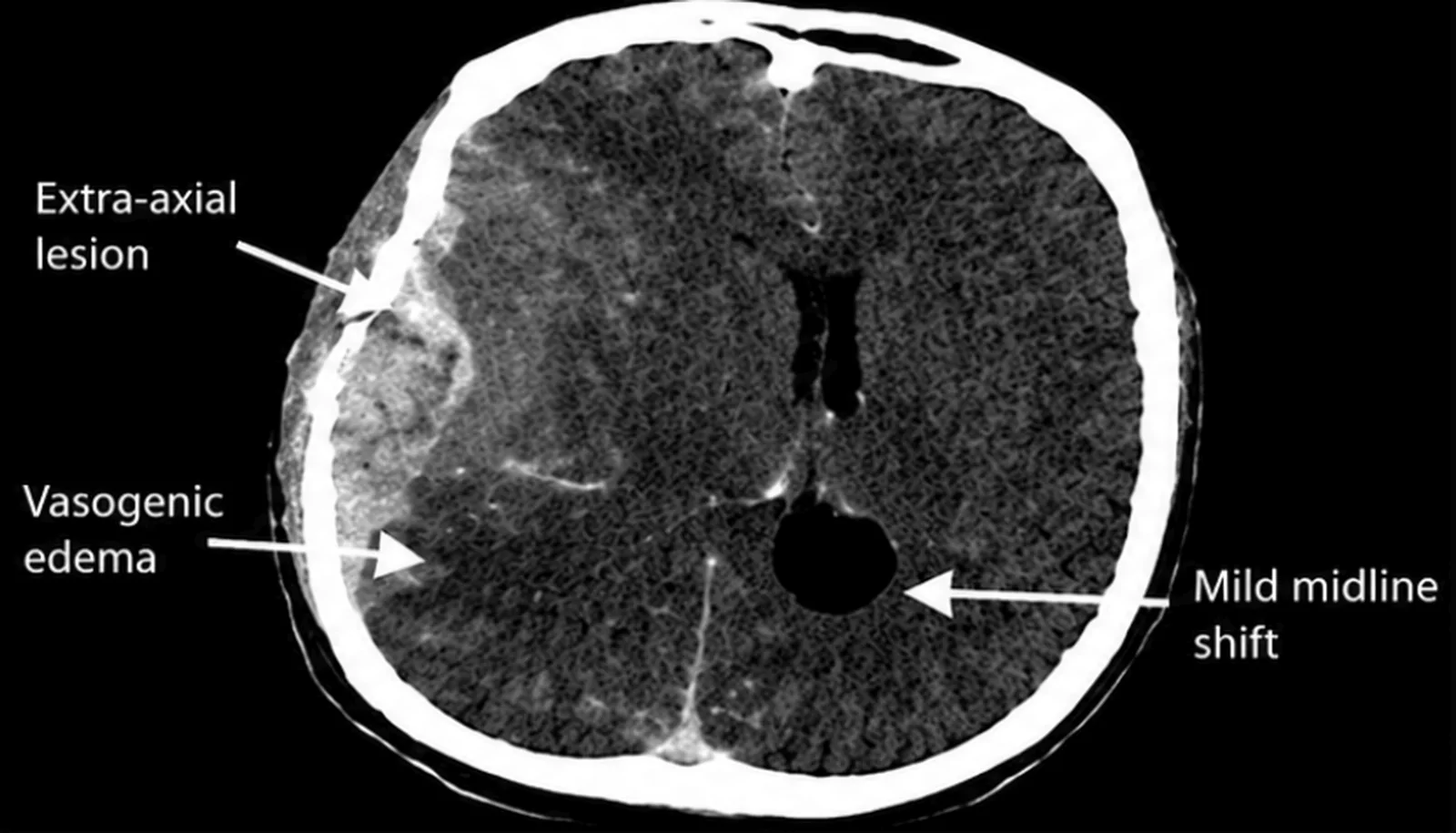
Preoperative contrast-enhanced CT scan. Axial contrast-enhanced computed tomography (CT) demonstrating a right frontotemporoparietal extra-axial lesion (white arrow) associated with extensive vasogenic edema (arrowhead) and mild leftward midline shift, initially suggesting a meningioma en plaque.

Contrast-enhanced cranial computed tomography (CT) demonstrated a large right frontotemporoparietal extra-axial lesion with broad dural implantation. The lesion exhibited both intracranial and extracranial extension, causing irregularity of the calvarium with invasion of the adjacent extracranial soft tissues. Extension to the sphenoid bone and the lateral wall of the right orbit was also observed (Figure 1).

**Figure 2:**
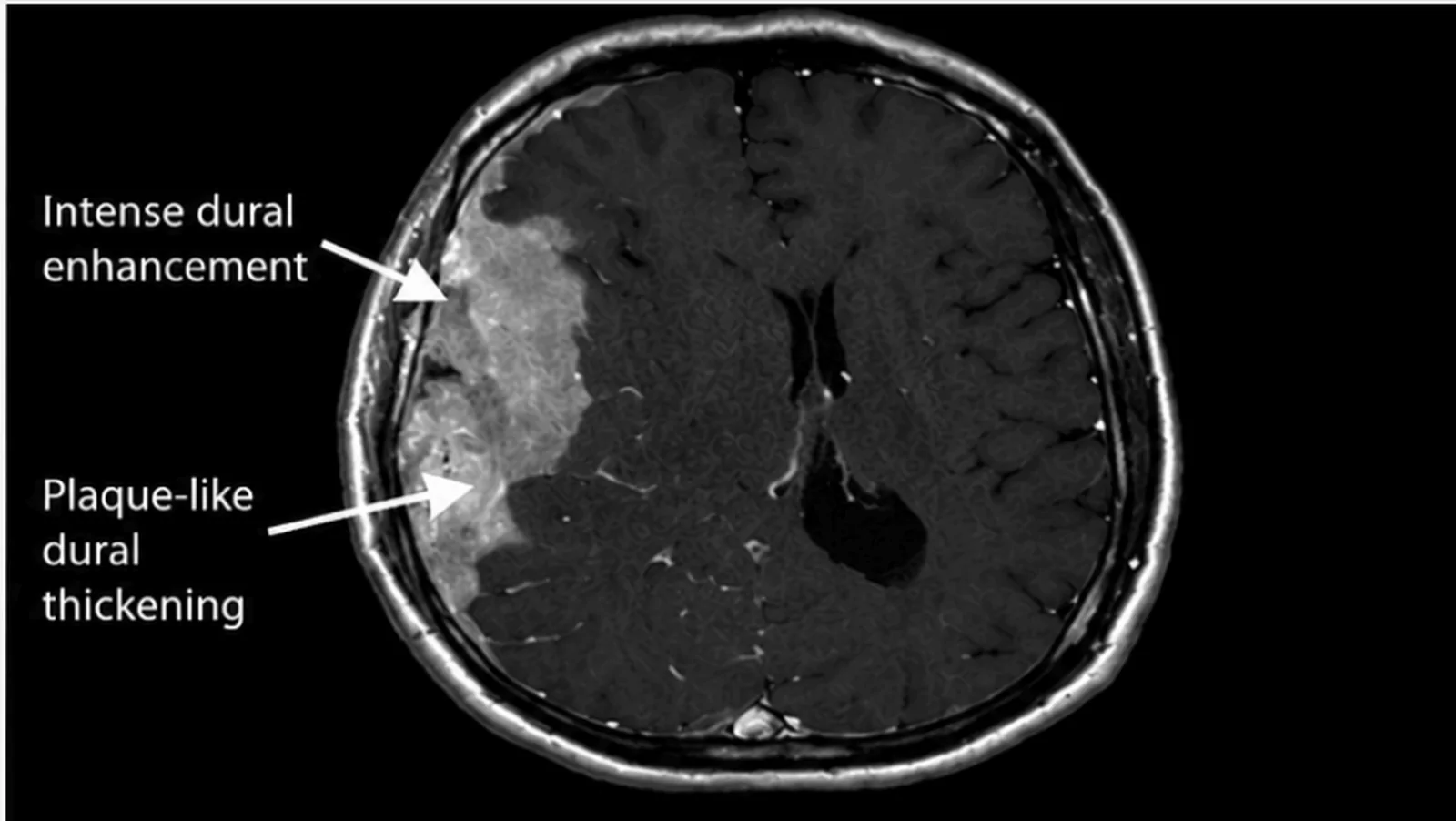
Preoperative contrast-enhanced T1-weighted MRI. Axial contrast-enhanced T1-weighted magnetic resonance imaging (MRI) demonstrating intense dural enhancement (white arrow) and plaque-like dural thickening (arrowhead), radiologically mimicking a meningioma en plaque.

**Figure 3:**
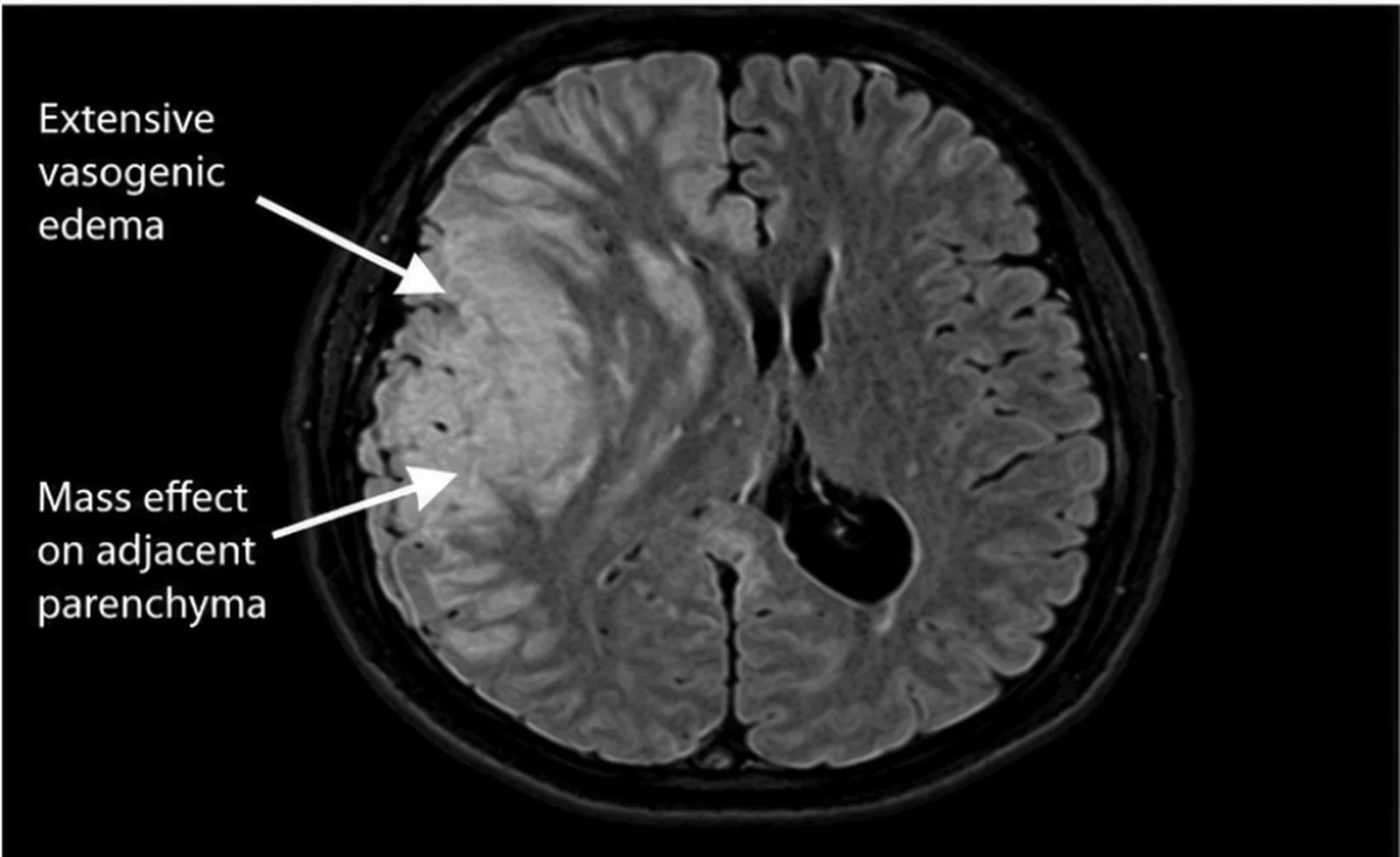
Preoperative FLAIR MRI. Axial fluid-attenuated inversion recovery (FLAIR) MRI demonstrating extensive surrounding vasogenic edema (white arrow) with significant mass effect on the adjacent brain parenchyma (arrowhead).

Staging CT scans of the chest, abdomen, and pelvis demonstrated diffuse osteolytic lesions consistent with disseminated metastatic disease. Brain magnetic resonance imaging (MRI) confirmed an extensive infiltrative lesion involving the cranial bone marrow and diploic space, associated with heterogeneous contrast enhancement and focal hyperintense areas on T1-weighted and FLAIR sequences (Figures 2-3). Based on the patient's oncological history and neuroimaging findings, the principal differential diagnoses included meningioma en plaque and dural metastatic disease.

Considering the patient's progressive neurological deterioration, significant mass effect, and the diagnostic uncertainty between meningioma en plaque and dural metastatic disease, microsurgical resection was recommended after multidisciplinary discussion. Although systemic staging demonstrated disseminated metastatic disease, surgery was undertaken with primarily diagnostic and palliative intent to obtain definitive histopathological diagnosis, relieve intracranial mass effect, and guide subsequent oncological management.

Complete excision of the infiltrated dura mater, involved calvarial bone, and infiltrated extracranial musculoskeletal tissues was achieved. During surgical exposure, approximately 1,700 mL of blood loss occurred because of the extensive infiltration of the surrounding soft tissues.

Intraoperatively, the lesion demonstrated diffuse pial invasion and shared vascular supply with the lateral aspect of the right cerebral hemisphere. Venous drainage occurred through the superficial Sylvian vein into the right sphenoparietal sinus, requiring meticulous microsurgical dissection to preserve adjacent vascular structures. Owing to the extensive dural defect, duraplasty was performed using galea aponeurotica and fascia lata grafts, followed by cranioplasty with polymethylmethacrylate bone cement.

**Figure 4:**
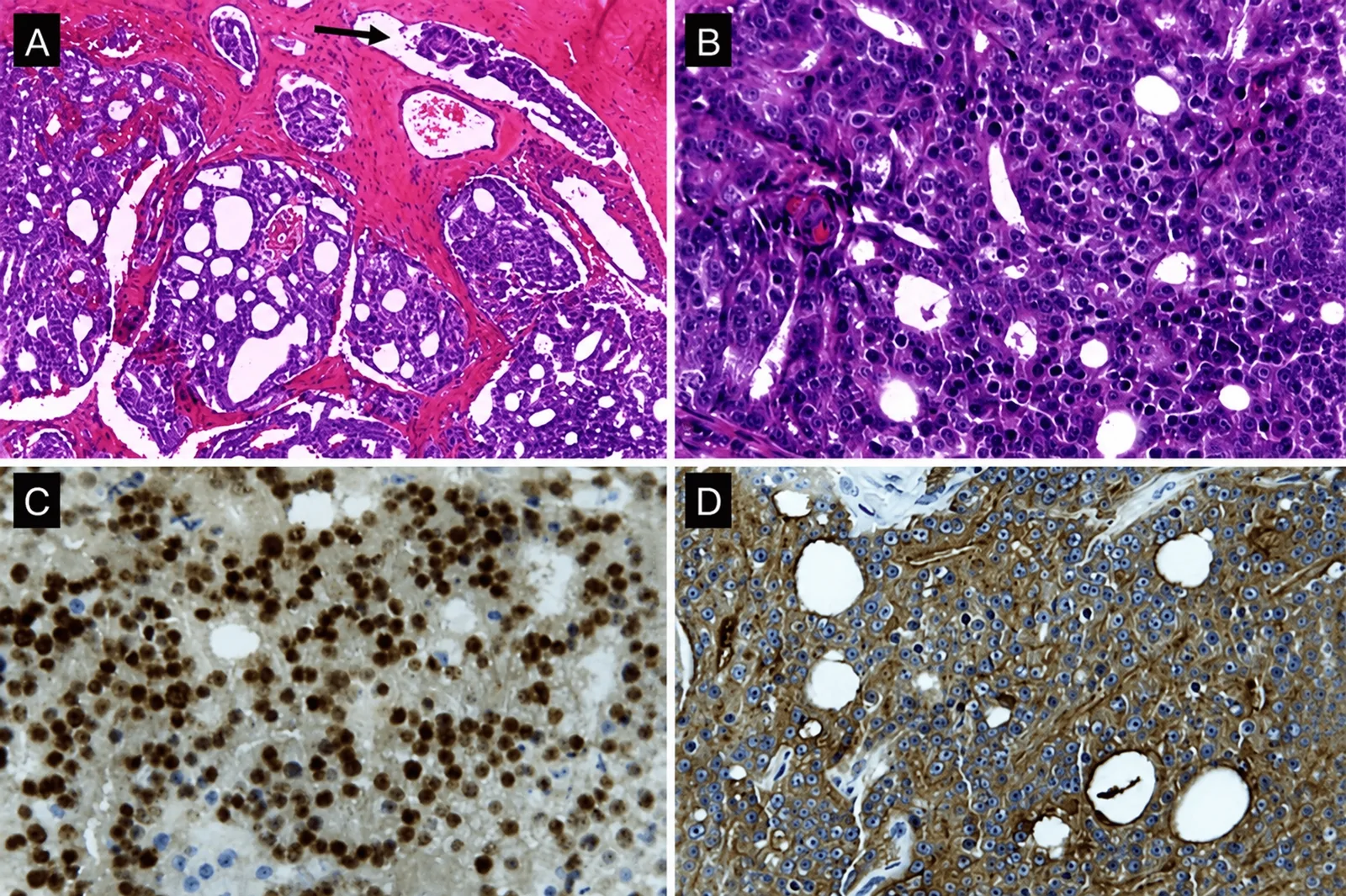
Histopathological and immunohistochemical findings confirming metastatic prostate adenocarcinoma. (A) Hematoxylin and eosin staining showing infiltrative adenocarcinoma with glandular architecture (original magnification ×100). (B) Higher magnification demonstrating glandular and solid growth patterns characteristic of metastatic adenocarcinoma (original magnification ×400). (C) Immunohistochemical staining demonstrating diffuse nuclear positivity for NKX3.1, confirming prostatic origin. (D) Immunohistochemical staining demonstrating diffuse positivity for prostate-specific antigen (PSA), supporting the diagnosis of metastatic prostate adenocarcinoma.

Histopathological examination demonstrated an infiltrative adenocarcinoma with glandular and solid architectural growth patterns on hematoxylin and eosin staining (Figures 4A-4B).

Immunohistochemical analysis revealed diffuse positivity for cytokeratin AE1/AE3, diffuse nuclear positivity for NKX3.1 (Figure 4C), and diffuse positivity for PSA (Figure 4D), whereas Napsin A was negative, confirming metastatic adenocarcinoma of prostatic origin and excluding the principal radiological differential diagnosis of meningioma en plaque.

**Figure 5:**
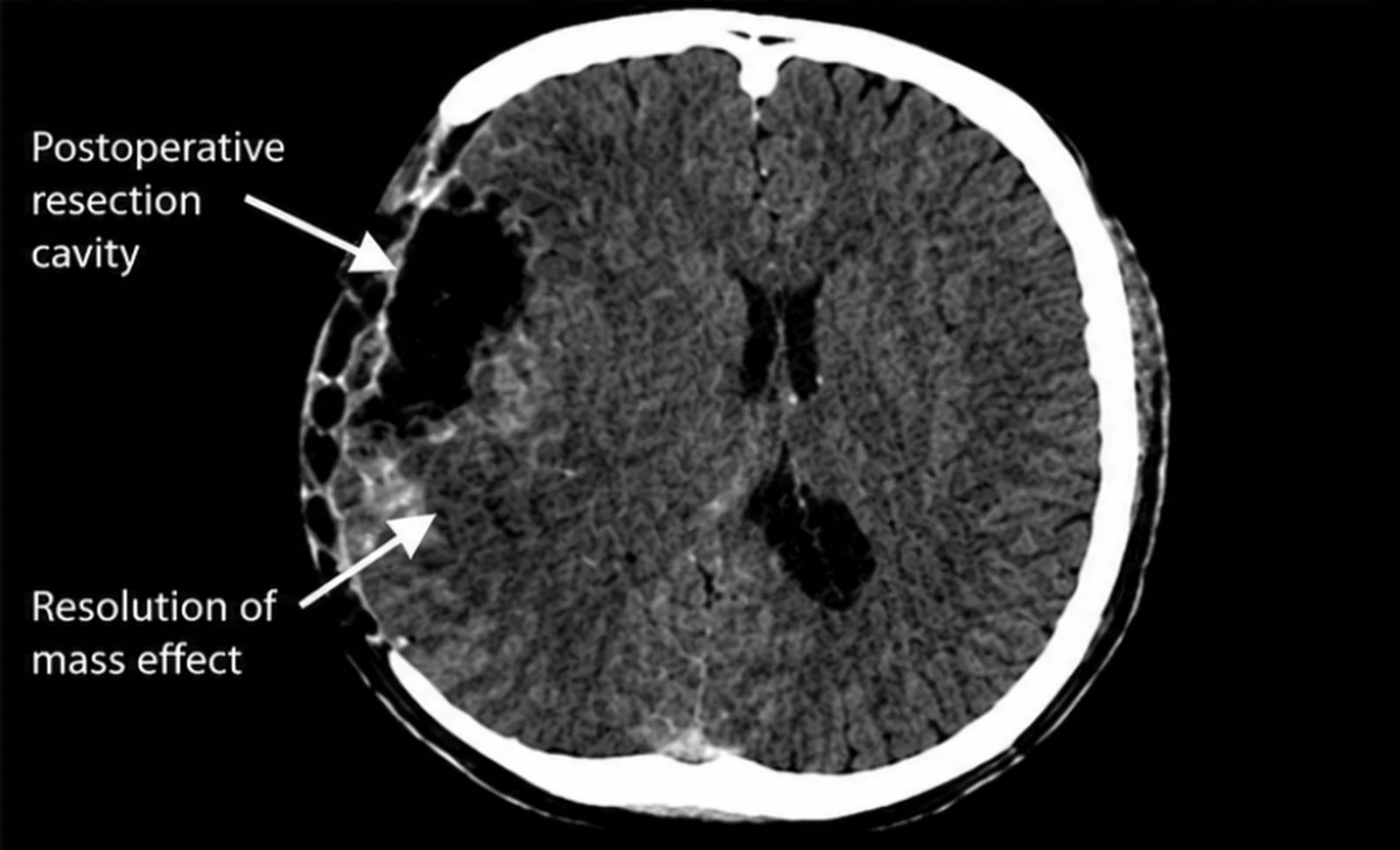
Postoperative CT scan. Postoperative axial computed tomography (CT) demonstrating the surgical resection cavity (white arrow) with marked reduction of the mass effect (arrowhead), consistent with satisfactory decompression following tumor resection.

Twenty-four hours after surgery, the patient developed acute neurological deterioration associated with anisocoria. Cranial CT demonstrated a right frontotemporal venous infarction complicated by extensive intraparenchymal hemorrhage (Figure 5). Despite maximal medical therapy, intracranial hypertension remained refractory, and the patient underwent emergency decompressive craniectomy with enlargement of the duraplasty and placement of an intraventricular intracranial pressure monitoring catheter.

Although intracranial pressure subsequently stabilized, right pupillary dilation persisted. Given the extensive systemic metastatic burden, the markedly elevated preoperative PSA level (1,020 ng/mL), devastating neurological injury, and poor overall prognosis, further aggressive treatment was considered unlikely to provide meaningful clinical benefit. After multidisciplinary discussion and shared decision-making with the patient's family, palliative care was instituted. The patient died four days after the second surgical procedure.

## Discussion

Dural metastasis from prostate adenocarcinoma is an uncommon manifestation of advanced prostate cancer and represents a significant diagnostic challenge because its clinical and radiological characteristics frequently resemble those of primary dural tumors, particularly meningioma en plaque [5,8-10]. Although improvements in systemic therapies have substantially prolonged survival among patients with metastatic prostate cancer, intracranial dissemination remains rare, occurring in fewer than 0.6% of cases [2-4]. Consequently, neurosurgeons are increasingly encountering uncommon metastatic lesions that were previously considered exceptional.

To better characterize this rare entity, we performed a systematic review according to the Preferred Reporting Items for Systematic Reviews and Meta-Analyses (PRISMA) guidelines [11]. PubMed and MEDLINE databases were searched in June and August 2025 using predefined Medical Subject Headings (MeSH) and Descritores em Ciências da Saúde (DeCS) related to prostate cancer, metastasis, and central nervous system involvement. Studies including adult male patients with histologically confirmed dural metastases from prostate adenocarcinoma were considered eligible. Review articles, duplicate publications, unavailable full-text manuscripts, and studies lacking original clinical data were excluded.

**Figure 6:**
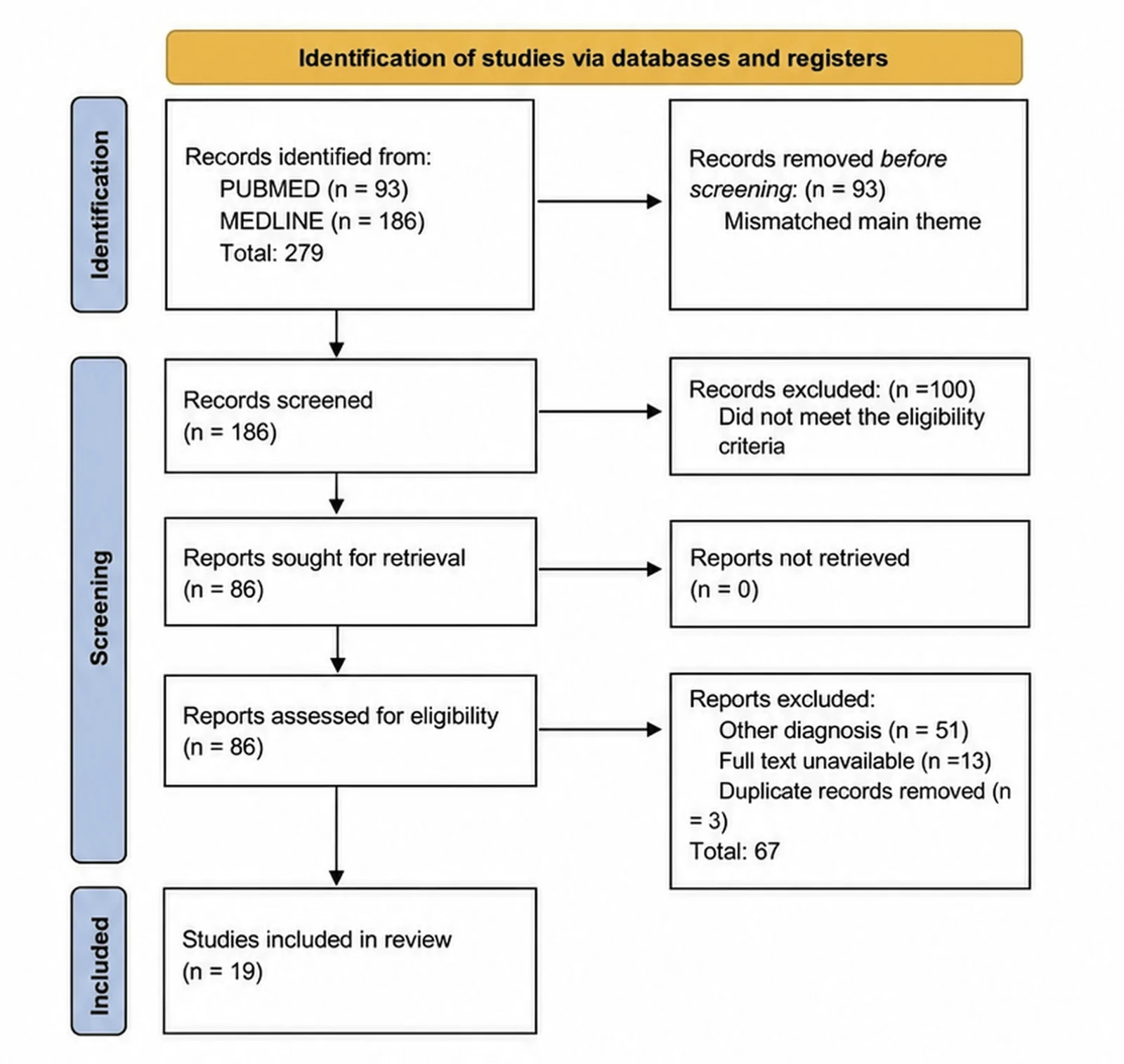
PRISMA flow diagram of the systematic review. Flow diagram illustrating the identification, screening, eligibility assessment, and final inclusion of studies evaluating dural metastases from prostate adenocarcinoma. A total of 279 records were identified through PubMed (n = 93) and MEDLINE (n = 186). Following screening and full-text eligibility assessment, 19 studies comprising 44 patients met the inclusion criteria for the systematic review.

A total of 279 records were initially identified. After duplicate removal, title and abstract screening, and full-text eligibility assessment, 19 studies comprising 44 patients met the inclusion criteria (Figure 6). The clinical, radiological, pathological, therapeutic, and outcome characteristics of the included studies are summarized in Table 1.

**Table 1:** Summary of published studies on dural metastases from prostate adenocarcinoma included in the systematic review. Abbreviations: HT, hormonal therapy; RT, radiotherapy; WBRT, whole-brain radiotherapy; SDH, subdural hematoma; NR, not reported.

Reference	Patients	Age (years)	Clinical presentation	Main imaging findings	Dural location	Treatment	Outcome
Ganau et al. [2]	20	48–78	Neurological deterioration	Various	Various	Surgery ± Gamma Knife ± RT ± HT	Mixed outcomes
Guedes et al. [5]	6	55–73	Cranial neuropathies, headache	Diffuse dural lesions	Multiple	NR	Mixed
Chen et al. [6]	2	70–71	Headache, seizures	Dural-based masses	Bilateral frontoparietal / Left frontal	Surgery + RT	One alive, one died
Yu et al. [7]	1	62	Upper limb weakness	Bilateral frontoparietal SDH	Bilateral frontoparietal	WBRT	Palliative
Alcalá-Cerra et al. [8]	1	60	Dysarthria, hemiparesis	Extra-axial enhancing lesion	Left frontotemporoparietal	NR	Palliative care
Cioni et al. [9]	1	65	Hemiparesis	Extra-axial lesion with SDH	Left perirolandic	NR	NR
Gunia et al. [10]	1	62	Headache	Skull and dural metastasis	Parietal	Surgery + RT	Improved
Cante et al. [12]	1	70	Cognitive decline	Leptomeningeal nodules	Left parietal/right middle cranial fossa	Chemotherapy + RT	Died
Guraya et al. [13]	1	67	Confusion	Olfactory groove meningioma-like lesion	Bilateral frontotemporal	Surgery + RT	Died
Mitchell et al. [14]	1	72	Seizure	Hemorrhagic falcine lesion	Falx	Surgery + RT + chemotherapy	NR
Nunno et al. [15]	1	64	Altered mental status	Bilateral SDH	Bilateral frontotemporal	Craniotomy	NR
Cobo Dols et al. [16]	1	54	Headache	Dural metastases + SDH	Calvarium	Steroids + WBRT	Died
Ferreira et al. [17]	1	60	Hearing loss	Subdural lesion	Right frontoparieto-occipital	Radical prostatectomy + HT + chemotherapy	Died
Olson et al. [18]	1	61	Cluster headache, diplopia	Meningioma-like lesion	Tentorium	Hormonal therapy + RT	Died
Zhang et al. [19]	1	87	Back pain	Intradural spinal metastases	Cauda equina	Hormonal therapy	Alive
Jodeh et al. [20]	1	60	Aphasia, cranial neuropathies	Diffuse pachymeningeal thickening	Diffuse	Hormonal therapy + chemotherapy + RT	Died
Dehghani et al. [21]	1	69	Headache, diplopia, visual impairment	Diffuse meningeal enhancement	Right occipital	WBRT	Died
Garousi et al. [22]	1	68	Progressive headache, fatigue	Diffuse meningeal thickening	Right occipital	WBRT + docetaxel + abiraterone	Alive
Michael et al. [23]	1	75	Vomiting, gait disturbance, cognitive decline	NR	NR	NR	Died

Surgical resection was the most frequently reported therapeutic approach, generally followed by radiotherapy, androgen deprivation therapy, or systemic chemotherapy when clinically feasible. However, this finding should not be interpreted as evidence supporting the superiority of surgery over alternative management strategies. The available literature is descriptive, lacks comparative studies, and is subject to substantial selection and publication bias, as surgically treated or diagnostically challenging cases are more likely to be reported than patients managed conservatively. Consequently, the optimal therapeutic strategy for dural metastases from prostate adenocarcinoma remains uncertain. Survival data were unavailable for nearly half of the published cases, further limiting interpretation of reported outcomes.

The present case shares several characteristics with previously reported patients. Neuroimaging demonstrated a large frontotemporoparietal extra-axial lesion associated with diffuse dural thickening, calvarial destruction, and extracranial extension, findings highly suggestive of meningioma en plaque. Such radiological overlap makes preoperative differentiation particularly challenging and frequently results in an initial diagnosis of a primary dural neoplasm [5,7,8,10,14,15]. In our patient, despite disseminated systemic disease, surgery was indicated because of progressive neurological deterioration, significant mass effect, and persistent diagnostic uncertainty. Therefore, the primary goals of surgery were to obtain definitive histopathological diagnosis and provide symptomatic relief rather than achieve curative treatment.

Definitive diagnosis relies on histopathological and immunohistochemical examination. In our patient, hematoxylin-eosin staining demonstrated infiltrative adenocarcinoma with glandular and solid growth patterns. Immunohistochemistry showed diffuse positivity for cytokeratin AE1/AE3, NKX3.1, and PSA, confirming the prostatic origin of the lesion, whereas Napsin A was negative. Although PSA and NKX3.1 are among the most reliable markers for confirming metastatic prostate adenocarcinoma, their interpretation should always be integrated with the patient's clinical history, imaging findings, and histomorphological features [5,10,14,15].

The role of surgery in patients with dural metastases should therefore be considered on an individual basis. Surgical resection may establish the definitive diagnosis, relieve mass effect, reduce intracranial hypertension, and improve neurological symptoms in selected patients. Conversely, in patients with advanced systemic disease, poor functional status, or limited life expectancy, alternative approaches such as stereotactic biopsy, radiotherapy, systemic therapy, or best supportive care may be more appropriate depending on the overall clinical scenario. Because current evidence consists predominantly of isolated case reports, no conclusions can be drawn regarding the comparative effectiveness of these treatment strategies [2-4,20].

Our patient experienced an unfavorable postoperative course characterized by venous infarction, extensive intraparenchymal hemorrhage, refractory intracranial hypertension, and death despite prompt surgical reintervention. Although poor outcomes have frequently been reported, the true prognosis of dural metastases from prostate adenocarcinoma remains difficult to determine because follow-up duration was inconsistently reported, outcome measures were heterogeneous, and survival data were unavailable for many published cases. Therefore, mortality observations should be interpreted cautiously rather than as definitive estimates of prognosis.

The principal limitation of the available literature is that the current evidence consists almost exclusively of isolated case reports and small case series. Publication bias, selection bias, heterogeneous reporting, and incomplete follow-up substantially limit the ability to determine the true incidence, natural history, optimal management strategy, and prognostic factors associated with this rare condition. Accordingly, the findings of the present review should be considered descriptive and hypothesis-generating rather than evidence supporting definitive clinical recommendations. Prospective multicenter registries and collaborative observational studies with standardized reporting of clinical, radiological, pathological, and outcome data are needed to improve understanding of the epidemiology, treatment strategies, and prognosis of dural metastases from prostate adenocarcinoma.

## Conclusions

Dural metastasis from prostate adenocarcinoma is a rare manifestation of advanced systemic disease that may closely mimic meningioma en plaque, making preoperative diagnosis particularly challenging. Histopathological examination with immunohistochemical confirmation remains essential for establishing the diagnosis when imaging findings are inconclusive. Our case highlights the importance of considering dural metastasis in the differential diagnosis of atypical dural-based lesions in patients with a history of prostate cancer. However, because the currently available evidence consists almost exclusively of isolated case reports and small case series, conclusions regarding the optimal management strategy should be interpreted with caution. Additional multicenter collaborative studies and prospective registries are needed to better define the clinical presentation, imaging characteristics, treatment selection, and outcomes of this rare condition.
